# Emerging role of cancer stem cells in the biology and treatment of ovarian cancer: basic knowledge and therapeutic possibilities for an innovative approach

**DOI:** 10.1186/1756-9966-32-48

**Published:** 2013-08-01

**Authors:** Federica Tomao, Anselmo Papa, Luigi Rossi, Martina Strudel, Patrizia Vici, Giuseppe Lo Russo, Silverio Tomao

**Affiliations:** 1 Department of Gynaecology and Obstetrics, University of Rome “Sapienza”, Rome, Italy; 2 Department of Medico-Surgical Sciences and Biotechnologies, University of Rome “Sapienza”, Latina, Italy; 3 Department of Medical Oncology, National Cancer Institute of Rome, Rome, Italy

**Keywords:** Epithelial ovarian cancer, Ovarian cancer stem cells, Chemoresistance, Target therapy

## Abstract

In 2013 there will be an estimated 22,240 new diagnoses and 14,030 deaths from ovarian cancer in the United States. Despite the improved surgical approach and the novel active drugs that are available today in clinical practice, about 80% of women presenting with late-stage disease have a 5-year survival rate of only 30%. In the last years a growing scientific knowledge about the molecular pathways involved in ovarian carcinogenesis has led to the discovery and evaluation of several novel molecular targeted agents, with the aim to test alternative models of treatment in order to overcome the clinical problem of resistance. Cancer stem cells tend to be more resistant to chemotherapeutic agents and radiation than more differentiated cellular subtypes from the same tissue. In this context the study of ovarian cancer stem cells is taking on an increasingly important strategic role, mostly for the potential therapeutic application in the next future. In our review, we focused our attention on the molecular characteristics of epithelial ovarian cancer stem cells, in particular on possible targets to hit with targeted therapies.

## Introduction

Epithelial ovarian cancer (EOC), a tumor originating from ovarian epithelial surface, includes different histological subtypes [1-3]. In 2013, there will be an estimated 22,240 new diagnoses and 14,030 deaths from this neoplaia in the United States [4,5].

It is the fifth most frequent cause of death from cancer in females and the most lethal cancer among gynecological tumors, with severe impact on public health and social costs [6-9]. Unfortunately, unlike other gynecologic cancers, etiology of EOC is still unkown [10]; and for biological and clinical reasons EOC is still diagnosed and treated at a very advanced stage; still now an early diagnosis is very difficult and infrequent and a validated program of screening for this tumor is still lacking [11-13].

Furthermore, despite the improved surgical approach and the novel active drugs that are available today in clinical practice, at the time of diagnosis about 80% of women have an advanced disease, with a 5-year survival rate of only 30% [12]; probably, one of the possible reasons could be the ovarian cancer cells ability to develop resistance mechanisms to the drugs through congenital and acquired genetic characteristics [14].

However, the response rate to chemotherapy is high, sometimes impressive and about the 75% of patients initially respond to conventional chemotherapy; moreover clinical and pathological complete remission is possible, sometimes frequent, but unfortunately >80% of these women experience relapse and die for the progression and diffusion of chemotherapy resistant disease. In fact, patients with recurrent EOC usually receive multiple lines of chemotherapy, with disappointing and unsatisfactory results, due to the occurrence of drug-resistant clones [15,16]. The pharmacological activity of drugs used in EOC could be reduced by a biological phenomenon that is able to induce the transformation of epithelial to mesenchymal cells (EMT) and the progression, invasion and diffusion of the tumor [17]. In the last years a growing scientific knowledge about the molecular pathways involved in ovarian carcinogenesis has led to the discovery and evaluation of several novel molecular targeted agents, with the aim to test alternative models of treatment in order to overcome the clinical problem of resistance. In this context the study of ovarian cancer stem cells (CSCs) is taking on an increasingly important strategic role, mostly for the potential therapeutic application in next future [18]. Now we know that self-renewing ovarian CSCs or ovarian cancer-initiating cells, and mesenchymal stem cells (SCs) too, are probably implicated in the etiopathogenesis of EOC, in its intra- and extra-peritoneal diffusion and in the occurrence of chemoresistance [19]. EOC can be classified into multiple types (serous, endometrioid, clear cell, and mucinous), with different clinical- pathologic properties, prognostic characteristics and therapeutic outcomes [20-22].

Moreover, several works support the fact that all histological cell types of EOC have different cellular origin with specific biologic and genetic profiles [23-27].

Consequently, the CSC population for each type may also be variable. It is therefore not surprising that SC properties have been reported in EOC cells isolated using different cell surface markers, including CD44, CD133 or CD24. Each of these EOC cells may represent either a hierarchy of CSC or an entirely different population of CSC for that particular ovarian histology [28-33]. CSCs support the succession of clonal tumor cell proliferation and repopulation in the tumor microenvironment. In fact they are predominantly quiescent, have up-regulated DNA repair capacity, are noncommittal to apoptosis and over-express ATP-binding cassette (ABC) drug efflux transporters and a profusion of cancer gene signatures [34,35].

The optimal management modality for EOC includes histopathological diagnosis and staging, surgical debulking of tumor, and the use of several cycles of chemotherapy with carboplatin and paclitaxel at maximum tolerated doses, eventually associated with bevacizumab, followed by maintenance or salvage treatments, in cases of disease recurrence [36]. Moreover, the majority of standard or experimental regimens have not yet improved efficacy or significantly reduced adverse effects over the combination of carboplatin, paclitaxel and bevacizumab, suggesting that other therapeutic experimental strategies are necessary in order to prolong progression free survival (PFS) and overall survival (OS) rates in ovarian cancer patients [37-43]. There is a critical need to develop broad-spectrum as well as individualized molecular-targeted therapies for EOC, and so current research interest is to identify signal transduction pathways and target key molecular role players that direct ovarian tumor sensitivity and resistance to therapy [44,45]. The aim of this review is to outline recent developments in our understanding of the interrelationships among selected ovarian CSC biomarkers, heterogeneous expression signatures and related molecular signal transduction pathways, and their translation into futuristic as well as more efficacious targeted treatment strategies.

### Cancer stem cell

A recent American Association for Cancer Research (AACR) workshop defined CSC as a malignant cancer cell with a stem cell phenotype [35]. Whilst the CSC hypothesis does not specifically address the mechanisms of malignant transformation, it has been suggested that CSCs are the malignant counterparts of normal adult tissue SCs which, due to dysregulated signaling pathways, are unable to maintain stem cell homeostasis. As well as the normal Scs, also CSCs are thought to reside at the top of the lineage hierarchy and give rise to differentiated cells, which themselves have no potential for self-renewal, and therefore do not contribute significantly to tumor growth. Due to their long life, SCs remain in a tissue for longer periods compared to their differentiated progeny, thereby making them more likely to acquire transforming mutations. Additionally, it is generally accepted that SCs are more resistant to apoptosis and DNA damage and they are therefore more likely to survive to any insults [46,47]. Whilst being quiescent in normal tissue, SCs are able to maintain their pool by undergoing asymmetric cell division during biological processes such as the occurrence of tissue damage. During this process, a SC divides asymmetrically to generate an identical daughter cell that is committed to differentiation. It has been suggested that in this way CSCs generate the different cell types within a tumor, leading to tumor self-renew as well.

Specific signaling pathways are involved in embryogenesis processes, leading to the development of various organs. We are talking about several key pathways, such as sonic Hedgehog, Notch, PTEN, BMI-1, WNT, and p53. During the development of cancer an alteration of these pathways occurs and this event could lead to dysregulation of SC self-renewal and contribute to tumor proliferation [19,48]. The SC pool is also tightly regulated by signaling pathways from the microenvironment of the SC niche, and several of these pathways, including Hedgehog and Wnt, have been implicated in carcinogenesis [49,50]. This may have very important implications in therapeutic interventions, including explanation for the development of chemoresistance. A role for CSCs in propagating and maintaining metastases has been proposed [51-54]. The different hypothesis that the dedifferentiation of mature cells to a more pluripotent state could be a potential mechanism for the development of SC-like features by cancer cells, cannot be dismissed. In 1997 Bonnet and Dick first isolated the CSCs in leukemic cells expressing SC marker CD34 and afterwards, also, in other solid tumors [55-64]. Classically, SCs are defined by their two main characteristics: self-renewal and pluripotency [63]. Experiments performed on human acute myeloid leukemia and solid tumors show that CSC have three functional characteristics:

transplantability, tumorigenic potential to form tumors when injected into nude mice;

distinct surface markers;

ability to recreate the full phenotypic heterogeneity of the parent tumor [64-66].

In characterizing normal and CSC s the problem is that these cellular populations are rare and the absence of specific cell surface markers represents a challenge to isolate and identify pure SC populations [67-72].

## Cancer stem cell markers

The limitation of using cell surface marker expression to characterize CSCs is that this approach requires prior knowledge of cell surface markers that are expressed by the putative CSCs in the tissue of interest, and often the choice of markers is inferred from known expression of markers in normal adult SCs. Several studies have prospectively isolated CSCs by looking for the presence of extracellular markers that are thought to be SC specific. The markers most commonly used are CD133 and CD44 [73]. These markers have been used successfully to isolate SCs in normal and tumor tissue [74,75]. Whilst CD133 and CD44 are thought to be indicative of a CSC phenotype, it is not clear if they are universal markers for characterizing CSCs derived from all types of tumors. Furthermore, expression of CD133 and CD44 may not be restricted to the CSC population and may be present in early progenitor cells.

The pentaspan transmembrane glycoprotein CD133, also known as Prominin-1, was originally described as a hematopoietic stem cell marker [73] and was subsequently shown to be expressed by a number of progenitor cells including those of the epithelium, where it is expressed on the apical surface [76]. Regarding EOC, Ferrandina G et al. demonstrated that CD133(+) cells gave rise to a larger number of colonies than those documented in a CD133(−) population. Moreover, CD133(+) cells showed an enhanced proliferative potential compared to CD133(−) cells. The percentages of CD133-1 and CD133-2 epitopes expressing cells were significantly lower in normal ovaries/benign tumors with respect to those in ovarian carcinoma. Both the percentages of CD133-1- and CD133-2-expressing cells were significantly lower in metastases than in primary ovarian cancer. They didn’t detect any difference in the distribution of the percentage of CD133-1- and CD133-2-expressing cells according to clinicopathologic parameters and response to primary chemotherapy. Using flow cytometry, Ferrandina et al. reported that CD133-1 and CD133-2 were both expressed in human ovarian tumors at higher frequency than in normal ovaries and metastatic omental lesions. CD133-1 and CD133-2 may be useful, therefore, to select and enrich population of CD133(+) ovarian tumor cells that are characterized by a higher clonogenic efficiency and proliferative potential [77].

Moreover, in 2009 Baba et al. found that *CD133* expression is repressed concomitant with the acquisition of DNA methylation in CD133− progeny of CD133+ cells supports a role for CD133 in the CD133+ cells, which is not required in the CD133− cells after asymmetric division [78].

According to these discoveries, Curley et al. found that tumor-derived CD133-1 cells have an increased tumorigenic capacity and are capable of recapitulating the original heterogeneous tumor [79].

Aldehyde dehydrogenase (ALDH), a reported CSC marker in several solid tumors, has been studied in association to CD133 in order to identify a set of markers to identify ovarian CSCs. Siva et al. discovered that the presence of ALDH(+)CD133(+) cells in debulked primary tumor specimens correlated with reduced disease-free and overall survival in ovarian cancer patients [31].

CD44 is a surface molecule which mediates cell adhesion and migration by binding extracellular matrix components such as hyaluronic acid, osteopontin, or activating receptor tyrosine kinases, which are related with tumor progression and metastasis [55,80]. Bapat et al. found that the growth factor receptors c-met and epidermal growth factor receptor were up-regulated in ovarian CSCs as well as CD44. They also expressed E-cadherin. Correspondingly, Snail, a known mediator of EMT through transcriptional repression of E-cadherin, was expressed in some CSC clones and to a lesser extent in others [22]. It has been demonstrated that CD44 + CD117+ cells are often present in EOC. CD117, beyond his role in cancer initiating cells from primary human tumors, has been used as stem cell marker for identification and characterization of hematopoietic stem and progenitor cells, of cardiac CD117-positive stem cells in adult human heart and other mesenchymal stem cells. Chen et al. demonstrated in vitro that human epithelial ovarian cancer CD44 + CD117+ cells possessed the properties of let the tumor be chemoresistant to conventional therapies, such as 5FU, docetaxel, cisplatin, and carboplatin [81].

CD44 has also been demonstrated to be associated with other CSC markers. In fact,Wei at al., investigating about Müllerian Inhibiting Substance with the aim of inhibit stem/progenitors in EOC, identified eight marker panel on three human ovarian cancer cell lines and found that the combination of Epcam^+^, CD24^+^, and CD44^+^ formed more colonies than other marker combinations. It was necessary to use this 3+ panel in combination, as each marker alone was not sufficiently selective [82].

Goodell et al. first reported a small population of cells showing distinct fluorescent-activated cell sorting profile off to the side of the main population due to a more efficient Hoechst dye efflux and lower fluorescent intensity signal. This subset of cells is referred to as the side population (SP) and is enriched for HSCs from murine bone marrow [83]. Many studies of SP have been performed in a number of cancers such as leukemias, brain, prostate, gastrointestinal tract, melanoma, retinoblastoma, and many cancer cell lines, leading to the hypothesis that the SP is enriched with CSC [84-90].

Szotek and colleagues investigated on several markers of SP and non-SP cells, such as c-kit/CD117, CD44, CD24, CD34, CD105, CD133, Sca-1, CD24, Ep-CAM. Taken together, all CSC surface markers investigated here are indicators, but definitely not a reliable marker for defining a population of CSCs in solid tumors since they do not characterize tumorinitiating cells exclusively. To increase the sensitivities and specificities for the detection of CSCs, further investigations are needed [91,92].

CD24 is a glycosylphosphatidylinositol-linked cell surface protein expressed in various solid tumors [93].

Gao et al. have successfully isolated CD24+ CSCs from ovarian tumor specimens and identified CD24 as a putative CSC marker in EOC [94]. The depletion and over-expression of CD24 could regulate the phosphorylation of STAT3 and FAK by affecting Src (non-receptor tyrosine kinases) activity. CD117, known as c-kit, is a type III receptor tyrosine kinase involved in cell signal transduction. It is involved in various cellular processes, including apoptosis, cell differentiation, proliferation, and cell adhesion [95]. High expression level of CD117 was observed in ovarian cancers [22].

Luo and his colleagues further demonstrated that CD117+ ovarian cancer cells had the ability to self-renew, differentiate, and regenerate tumor compared to CD117- in xenograft model [96]. It has been also suggested that CD117 in ovarian carcinoma was associated with poor response to chemotherapy [97].

The activation of Wnt/β-catenin-ATP-binding cassette G2 pathway was required for cisplatin/paclitaxel-based chemoresistance caused by CD117 in ovarian CSCs [98].

The epithelial cell adhesion molecule EpCAM is a glycosylated membrane protein. It is highly expressed in different tumor types, including colon, lung, pancreas, breast, head and neck and ovary [99]. EpCAM was found to be hyperglycosylated and frequently associated with cytoplasmic staining in carcinoma tissues [100]. EpCAM is comprised of an extracellular domain (EpEX), a single transmembrane domain and a short 26-amino acid intracellular domain (EpICD). Among them, EpEX is required for cell-cell adhesion [101]. Down-regulation of EpCAM could cause loss of cell-cell adhesion and promote EMT [102,103].

A valid marker among several malignant and non malignant tissues is aldehyde dehydrogenase-1A1 (ALDH1A1). ALDH1A1 is an intracellular enzyme that oxidizes aldehydes; it has a detoxifying role, and allows the conversion of retinol to retinoic acid, making a management of the differentiation pathways. It holds the attractive distinction of not only being a potential marker of “stemness,” but potentially playing a role in the biology of tumor initiating cells as well [104-110]. ALDH1A1 was identified as a putative CSC marker, and it was associated with chemoresistance in the ovarian CSC [111].

In one case, clones have been identified from tumor ascites; they were able to form anchorage-independent spheroids and have shown to express the SC markers Oct ¾, Nanog and the progenitor marker Nestin [112].

Szotek et al. used flow cytometry to isolate a SP of cells from genetically engineered mouse ovarian cancer cell lines that expressed the multidrug transporter protein BCRP1 and were resistent to doxorubicin, suggesting a possible link between CSCs and chemoresistance. They also isolated a similar smaller SP of cells from the human ovarian cancer cell lines IGROV-1, OVCAR3, and SKOV3, but these SP cells were not further characterized [91]. Two other studies have independently defined ovarian cancer SC by evaluating CD44+ CD117+ and CD133+ phenotypes. The latter suggests an epigenetic regulation of the CD133 promoter [22,29]. Additionally, using CD44, stem-like cells were enriched from patients’ samples and were characterized by Myd 88 expression and chemokine and cytokine production [20]. Despite the different profiles described for CSCs by these studies, both studies reported that the CSC phenotype was more resistant to platinum based therapy, which again supports the theory that CSCs may be responsible for chemoresistance. Generally, these studies highlight the lack of consensus about the molecular characteristics of ovarian CSCs. It is likely that the expression of markers overlaps and both CD133 and CD44 characterize the ovarian CSC. Alternatively, there may be more than one population of cells with SC properties in ovarian cancers. The study by Bapat et al. postulated that SCs are the target of transformation in ovarian cancer because few clones isolated from ovarian cancer ascites spontaneously immortalized in culture, suggesting a model for disease development. In their study, about mutant mitochondrial genome, Wani et al. highlighted the importance of tumor status suppressor gene - cAMP responsive element binding protein (CREBBP); in fact the mutation of this gene could be used by a normal SC to overcome the DNA repair in the its evolution towards tumorigenesis [113].

Other mechanisms leading to SC enrichment under conditions of stress include heightened DNA damage response and repair, both contributing significantly to tumor survival [114,115].

## Resistance to conventional therapies

Although the standard combination of surgery and chemotherapy can effectively reduce tumor mass, most patients, eventually with residual ovarian CSCs, acquire chemoresistance [116-121]. The CSC theory supports that even if a small number of CSCs remains in situ after therapy, disease recurrence can occur [19]. Several pathways could be involved in these mechanisms including activation of anti-apoptotic factors, inactivation of pro-apoptotic effectors, and/or reinforcement of survival signals [122]. Based on an understanding of their characteristics, the refractory response of CSCs to drugs and radiation treatments may be attributed to:

•drug effluxion

•glutathione (GSH) system

•apoptosis;

•enrichment of CSCs during disease progression

•tumor dormancy and CSC quiescence

## Drug effluxion

It can be caused by an altered uptake or efflux of drug in the target cell. Platinum compounds enter the cell, primarily by passive diffusion, however several different ways have been described such as copper transporter proteins (CTR), organic cation transporters (OCTs) from de SLC22 family, ATP-binding cassette (ABC) multidrug transporters, copper-transporting ATPases, and multidrug and toxin extrusion from the SLC47 subfamily members that might facilitate the active efflux of anticancer platinum agents. Some of most frequently studied drug transporters associated with acquisition of resistance in normal SCs as well as in CSCs are multifunctional efflux transporters from the ABC gene family [123]. These contribute to tumor resistance by actively transporting drugs across cell membranes through ATP hydrolysis [83,124-127]. Efflux transporters in the ABC family such as ABCG2 are cell surface drug-resistance markers involved in the transport of substances and cellular products [128-133]. The resistance gene BCRP/MXR/ABCP has been studied for its involvement in development of chemoresistance. ABCG2/BCRP plays a key role in cellular homeostasis and tissue integrity. It has been observed that ovarian CSCs exposed to chemotherapy overexpress this ABC family of transporters. Consequently, ABCG2/BCRP acts as a xenobiotic drug transported by promoting expulsion through an ejection system.

## Glutathione (GSH) system

Also inflammatory processes can contribute to multiple CSC capabilities by supplying bioactive molecules to the tumor microenvironment and, additionally, inflammatory cells can release reactive oxygen species that are actively mutagenic for nearby cancer cells and accelerate their genetic evolution toward states of heightened malignancy [134].

GSH system protects cells against the effect of external cytotoxic agents, including platinum [135,136]. The GSH system can suppress oxidative stress and maintain cellular redox homeostasis [137]. The contribution of GSH and GSH-related enzymes to chemoresistance has been demonstrated in different types of tumor, including ovarian cancer and brain tumor [138]. GSH is also involved in the detoxification of various xenobiotics [139]. Upon metabolism of chemotherapeutic agents, the enzymes of glutathione-S-transferase (GST) family could prompt the formation of GSH-drug conjugates. Many chemotherapeutic agents have been shown to conjugate with GSH, including chloroethylnitrosoureas, platinum compounds, and other alkylating agents. The resulting GSH drug conjugates are more water soluble and less active than the compounds themselves. They are thus exported from the cell via the transporter-mediated system [140].

Basing on these data we can affirm that GST can lead to a loss of response to chemotherapeutic agents, including those that are usually employed in the treatment of ovarian cancer. Some authors let us think that this is particularly significant in cancer cells with stem-cell like properties. In a recent study, it has been shown that platinum-resistant human cancer cells with stem-cell like EMT properties, had high cellular GSH and accumulated significantly less cellular platinum compared to their parental cells, and failed to undergo apoptosis when exposed to platinum at the drug concentrations toxic to the parental cells [140].

## Apoptosis

Apoptosis can condition response to antitumor drugs and it’s regulated by several molecular phenomena, such as the expression of Bm-1 and the loss of p53.

Bmi-1, a member of the polycomb group (PcG) family, participates in the self-renewal and maintenance of CSCs [141]. As an oncogene, Bmi-1 could enable cancer cells to escape apoptosis by modulating multiple growth signaling pathways [142]. Thus, its overexpression in cancer cells could be used as a survival marker. The role of Bmi-1 in chemoresistance has been addressed recently [143,144]. For ovarian cancer cells, silencing of Bmi- 1 gene could promote sensitivity to cisplatin and induction of apoptosis [145].

The tumor suppressor gene p53 plays a critical role in cell proliferation and apoptosis by controlling several signaling pathways. In addition, the control of intracellular localization of p53 is also associated with the regulation of apoptosis and chemosensitivity in human ovarian cancer cells [146-148]. Loss of p53 function correlates with multidrug resistance in several tumor types, including EOC [149].

## Enrichment of CSCs during disease progression

Enrichment of CSCs in tumor tissues is reported in patients with response to therapy through mechanisms such as enhanced DNA damage repair and changes in the cellular phenotype between epithelial and mesenchymal states of cell [150]. EMT is a physiological transcriptional reprogramming event and is characterized by the combined loss of epithelial cell junctions and cell polarity and the gain of a mesenchymal phenotype. EMT and mesenchymal to epithelial transition (MET) processes are now recognized in cancer progression [151]. A link between CSC and EMT has been suggested, whereby transformed human mammary epithelial cells, that have undergone EMT, show a gain of the CSC phenotype [152-155].

Recently, Kurrey et al. have reported a detailed study of genome-wide identification of SNAI1 and SNAI2 targets that resolves the specific mechanism underlying enrichment of stem-like cells post radiation treatment or chemotherapy through EMT [156]. This study identifies a modulation by SNAI1 and SNAI2 toward repressing a newly acquired subset of gene targets under conditions of stress that results in inactivation of p53-mediated apoptosis. This tripartite functioning in which EMT mediates the escape mechanism to newer and less adverse niches,complemented with resistance to apoptosis and acquisition of ‘stemness’, ensures cell survival under conditions of stress and/or ensures tumor generation that correlates with disease progression. This suggests that such de novo CSC generation arises from a directed de-differentiation of tumor cells that culminates in selective accumulation of quiescent or resistant cells under conditions of stress. EMT confers the ability to detach from the primary bulk by losing cell adhesive properties and acquire invasive features to cancer cells. Furthermore, cancer cell populations, experimentally induced into EMT, exhibit an increased resistance to chemotherapy and acquisition of SCs properties [157].

## Tumor dormancy and CSC quiescence

Many CSCs stay in G0 and are not susceptible to cell cycle-specific chemotherapeutic agents [158]. Consequently, this sub-population could survive to such treatments and later it is able to regenerate the tumor [159]. However, as described above, the immunity and resistance that occur in CSCs are mainly due to genetic and epigenetic changes, that accumulate mutations and lead to the loss of apoptosis control. These changes include over expression of DNA repair protein MGMT and genes that reduce apoptosis process leading to invasion and metastasis in more advanced stages, including FLIP, Bcl-2, Bcl-XL, HER2/neu and numerous IAP family members. Altered Bcl-2 expression can drastically change drug sensitivity and is associated with resistance to multiple drugs in human cancers such as EOC [160]. Overexpression of proto-oncogene HER2, which encodes a trans-membrane phosphoglycoprotein receptor tyrosine kinase (p185HER2), constitutes an important step in progression, poor prognosis, and clinical outcome of ovarian carcinoma. This event can lead to malignant transformation and plays a crucial role in the tumorigenesis of ovarian cancer. Tumors with high HER2 expression show less sensitivity to anticancer drugs [161-163]. The cell could also maintain its drug insensitivity using epigenetic changes [164]. Thus, CSCs have characteristics that make them responsible for development of chemoresistance in both refractory and recurrent EOC. Hypoxia is another critical factor for cancer malignancy and maintenance of SC characteristics [165-168]. The hypoxia response system acts pleiotropically to up-regulate glucose transporters, mainly GLUT1, and multiple enzymes of the glycolytic pathway [169,170]. Glycolytic metabolism is associated with activated oncogenes and mutant tumor suppressors.

Multiple ovarian cancer cell lines have been studied in a recent analysis, and in taxane and platinum resistant cell lines; in this study the ALDH1A1 expression and activity were found to be significantly higher. Among patients, 72.9% of ovarian cancers had ALDH1A1 expression and the percent of ALDH1A1-positive cells correlated negatively with PFS [112].

## Therapeutic approaches of ovarian CSCs

Targeting CSCs might be a strategy to improve outcome of cancer patients but the complexities that lie within this approach will provide many challenges in clinical applications. Combined treatments that target CSCs will be a new direction in the future. Some of these hurdles include overcoming the immune heterogeneity in CSC population as well as the problem of epitopes shared with normal SCs and the necessity to identify additional CSCs antigens. Nevertheless, drug treatment for CSCs may increase the risk of toxicity since CSCs share common features with normal SCs. The current therapeutic strategies in ovarian CSCs are discussed below.

### Target therapy: cell surface markers

Antibody therapies against tumor cell surface antigens have improved clinical prognosis through inhibition of specific signaling pathways, enhancing activation of direct immune effectors. In some cases, antibodies are conjugated with a bioactive drug that enables selective targeting of chemotherapeutic agents. In other cases, they block a signaling pathway in which the marker may be involved.

A monoclonal murine anti-human CD133 antibody conjugated to monomethyl-auristatin F (MMAF), a potential cytotoxic drug, has been shown to inhibit growth in hepatocellular and gastric cancer cells in vitro by inducing apoptosis [171].

Several antibodies against CD44v6 isoform have been developed and phase I clinical trials for patients suffering from head and neck squamous cell carcinoma began with high hopes [20,172]. CD44 is a surface adhesion molecule that binds to hyaluronic acid, which is related with tumor progression and metastasis. Hyaluronic acid bioconjugates with paclitaxel are being studied to enhance selective entry of cytotoxic drugs into human EOC cells expressing CD44 and for its use in intraperitoneal treatment of ovarian carcinoma [173].

SWA11, an antibody against CD24,reduced tumor size in xenograft mice transplanted by lung cancer cells A549 and pancreatic cancer cells BxPC3 [174].

In 2009, Su and his colleagues successfully applied short hairpin RNA (shRNA) to reduce CD24 expression. The knockdown of CD24 decreased cell viability by in vitro activation of apoptosis in ovarian cell line SKOV3, also suppressing tumor growth in nude mice bearing ovarian cancer in vivo [175]. Therefore, CD24 inhibition may be considered as an effective approach for cancer therapy.

Imatinib, a potent CD117 (c-KIT) specific inhibitor, has been used in clinical trials for the treatment of many types of cancer, including persistent epithelial ovarian cancer [176].

c-KIT is a receptor tyrosine kinase involved in cell signal transduction. It has been also suggested that CD117 in ovarian carcinoma was associated with poor response to chemotherapy. Therefore, c-KIT could be a perfecttherapeutic target of a tyrosine kinase inhibitor as imatinib. Furthermore, Patel and his colleagues demonstrated that imatinib mesylate is involved in complex cellular processes, including metabolic pathways, cell cycle, cell proliferation, apoptosis, and signal transduction through mass spectrometry-based proteomics method in human ovarian cancer cell line A2780 [177].

EpCAM positive cells also have tumor-initiating potential, making it a potential target for cancer therapy. Catumaxomab, a monoclonal antibody against EpCAM is a trifunctional antibody, which can bind three different cell types, including tumor cells, T cells, and accessory cells (dendritic cell,macrophages, and natural killer cells) [178].

It is now used in phase III clinical trials in patients with malignant ascites [179].

The investigation of its efficacy and safety was also explored in phase II clinical trials evaluating advanced ovarian cancer patients who had experienced complete chemotherapy. Based on both preclinical and clinical outcomes, EpCAM may be served as a possible therapeutic target against epithelial ovarian cancer.

ALDH proteins are a superfamily containing 19 enzymes that protect cells from carcinogenic aldehydes [180]. Recently, clinical trials have been initiated using disulfiram (an ALDH inhibitor). The combination of disulfiram with gemcitabine had a synergistic effect on cytotoxicity in glioblastoma multiforme cells [181].

Targets such as CD133 and CD44 could differentiate CSCs from normal cells enabling specific action but indirect strategies,such as interfering with the establishment of an appropriate niche through anti-angiogenic or anti-stromal therapy, could be more effective.

### Target therapy: differentiation of CSCs

One way to treat cancer without removing CSCs is the induction of the differentiation and the loss of their self-renewal property. Drugs such as retinoic acid or drugs that aim to generate epigenetic changes in the tumor can stimulate CSCs differentiation. In any case, differentiation strategies might impact on proliferation rate, tumoral composition, self-renewal property, and phenotype trans-differentiation.

Retinoic acid and its analogs are the only differentiating agents used because they are modulators of differentiation and proliferation of epithelial cells. Their combined use with chemotherapy has proven to be a good method for treatment of acute promyelocytic leukemia [182,183]. The all-transretinoic acid (ATRA) can inhibit the proliferation and induce the differentiation via inhibition of Wnt/β-catenin pathway in head and neck squamous carcinoma CSC [184].

Recently, Whitworth and his colleagues effectively reduced the growth of ovarian CSC with carboplatin combined with three novel retinoid compounds [185]. In addition, specific unsaturated fatty acids (palmitoleic, oleic, and linoleic acids) can trigger adipocyte-like differentiation in many types of cancer cells, including ovarian cancer cell line SKOV3 [186].

In 2012, Yin and his colleagues observed that TWIST-1 (a basic helix-loop-helix transcription factor) played a key role in triggering differentiation of EOC [187].

Jain et al. recently reported that p53 (capable for regulating molecular networks) can activate two miRNAs (miR-34a and miR-145). These miRNAs were then shown to prompt differentiation of human embryonic stem cells [188]. Indeed, emerging evidence indicated that miRNAs were involved in self-renewal and differentiation of normal and cancer stem cells. It was suggested that such miRNAs should be a new therapeutic target for cancer treatment [189]. However, more detailed regulation of differentiation remains to be determined.

### Nanoparticles

Therapeutic nanoparticles (TNPS) consist of a therapeutic element, such as small-molecule drugs, proteins, or peptides, combined with a drug-delivery molecule, such as a polymers or lipids [190]. Given the high rate of recurrent ovarian cancers with chemotherapeutic resistance, the potential for a more efficient and direct delivery system provided by TNP’s size and versatility, makes them a potentially proficient treatment system. Five features are defined as being distinguishing for TNPs, and three of them are particularly relevant in treatment of recurrent ovarian cancer. First, their ability to carry a high drug payload without affecting the carrier molecules or ability of the nanoparticle to maneuver itself within tumor tissue, gives them an advantage over antibody conjugated to a targeting ligand. Second, the drug-delivery molecule can be customized to influence the speed of drug release of each specific drug it carries. Finally, TNPs utilize the enhanced permeability and retention (EPR) effect provided by immature, leaky tumor vasculature to localize tumor tissue. TNPs may be endocytosed by target cells, thereby bypassing mechanisms of resistance such as cell-surface protein pumps. The joint effort of the EPR effect and endocytosis method of targeting tumor cells provides a possible twofold benefit in cancer treatment. This approach minimizes side effects of widespread drug delivery and contributes to overcome resistance mechanisms, such as cell-surface protein pumps. In addition to anti-cancer drug delivery, controlled and targeted release through the EPR effect,combined with surface modifications, allow a direct interface with specific CSCs by utilizing particular surface markers, receptors, epitopes, or any other unique features of the CSCs, absent in healthy tissues and normal stem cells. The current TNPs used for ovarian cancer treatment are liposomal doxorubicin, xyotax (or CT-2103), and IT-101. This group of TNPs can be further separated into two groups based on the type of carrier molecule utilized. Liposomal doxorubicin differs from the other two using pegylated liposome molecule as its carrier molecule combined with doxorubicin. The second group consists of Xyotax and IT-101 that utilize polymeric carriers. Xyotax is a combination of poly-L-glutamic acid (PGA) and paclitaxel. IT-101 consists of a cyclodextrin containing polymer combined with camptothecin. One of the advantages that polymer-based TNPs have over lipid-based TNPs is that polymer-based TNPs are able to generate a more controlled drug delivery. The use of TNPs for each of these drugs allows lower drug clearance and a longer half-life [191]. In an in vivo orthotopic mouse model of ovarian cancer, ALDH1A1 silencing using nanoliposomal siRNA sensitized both taxane- and platinum-resistant cell lines to chemotherapy, significantly reducing tumor growth in mice,compared to chemotherapy alone. These data demonstrate that the ALDH1A1 subpopulation is associated with chemoresistance and outcome in ovarian cancer patients, and targeting ALDH1A1 sensitizes resistant cells to chemotherapy. ALDH1A1-positive cells have enhanced, but not absolutely, tumorigenicity, but do have differentiation capacity lacking in ALDH1A1-negative cells [112].

Niches of CSCs: Niches are microenvironments where CSCs reside, containing cell-cell, cell-extracellular matrix, and soluble factors that support the growth, progression, and metastasis of CSCs [192]. Bone-marrow-derived mesenchymal SCs (MSCs) are known to form fibroblast and myofibroblast populations in the tumor-associated stroma. Recently, evidence has been demonstrated that MSC and derived cell types could secrete prostaglandin E2 and release various cytokines, which are vital for the formation and progression of a tumor [193]. Furthermore, MSC affected metastatic ability and chemoresistance in two ovarian cancer cell lines: OVCAR3 and SKOV3 [194].

Katz et al. reported that tumorigenic ability of ovarian tumor cells was dependent on niches derived from human embryonic stem cells [195]. The hypoxic niches were beneficial for acquirement of stem-like properties of ovarian cancer cells [196]. These findings highlight the vital role of CSCs niches, which represent a promising therapeutic target for eradicating CSCs in the future. Indeed, disrupting components in the niches may yield better outcomes without noncytotoxic effect, when compared with that of removing the CSCs [197].

### MicroRNAs (miRNAs)

MiRNAs are a group of small noncoding RNAs with 20–28 nucleotides in length. They could regulate gene expression at post-transcriptional level. Thus, miRNAs are involved in diverse biological processes, such as development and tumorigenesis [198].

The expression profile of miRNAs was different between normal SCs and CSCs [199,200].

MiR-214 was highly expressed in ovarian CSCs and endowed the property of self-renewal and chemoresistance in ovarian CSCs via repressing p53- Nanog pathway [201].

MiR-199a significantly rescued the sensitivity of ovarian CSCs to some chemotherapeutic agents, including cisplatin, paclitaxel, and Adriamycin. Moreover, miR-199a prevented tumorigenesis in xenograft model via downregulating expression of CSCs marker CD44. In addition, the expression of miR-200a could reduce migrating ability of CD133+ ovarian CSCs. This was because miR-200a inhibited E-cadherin and ZEB2, two genes critical for migration process. However, some miRNAs own oncogenic property, such as miR-125, miR-9, miR-30, miR-21 and miR-215 [202,203].

## Discussion

Ovarian CSCs are likely to be heterogeneous as well as the EOC itself. Because of its semi-solid character in dissemination and growth, advanced EOC with its hundreds of peritoneal tumor nodules and plaques, appears to be an excellent in vivo model for studying cancer stem cell hypothesis. Until now, no universal single marker has been found to faithfully isolate ovarian CSCs. We can say that, even in multi-passaged cancer cell lines, hierarchic government of growth and differentiation is conserved and that the key CSC population may be composed of small overlapping cell fractions defined by various arbitrary markers.

The high rates and patterns of therapeutic failure seen in patients with EOC are consistent with a steady accumulation of platinum-resistant CSCs. We can say that targeting pathways, involved in this process, could significantly increase tumor sensitivity to platinum therapy, leading to novel treatment strategies upon diagnosis of EOC and recurrence [204-208].

An ideal agent should be able to selectively target CSCs over normal SCs. Without this selectivity, the effectiveness of treatment might be limited by systemic toxicity. It is also likely that treatment of patients with CSC-targeted therapies will require new clinical end points for monitoring therapeutic efficacy. These therapies in fact target only a small fraction of cells within the tumor, not the bulk of tumor. In addition, responses may require a much longer time so that they are typically visible. Rational approaches might also include the use of cytotoxic chemotherapies to target proliferating bulk of tumor in addition to CSC-directed therapy. An important end point would be to control the disease status by checking the size of the CSC population in response to treatment. In this area one strategy could be monitoring the burden of CSCs in circulation.

Microarray and proteomic profiling of CSCs will likely lead to identification of new markers, as well as potential therapeutic targets. CSC markers may have prognostic value by allowing assessment of the size of the CSC population within any selective tumor. Animal transgenic and xenografts model systems described above need to be implemented in order to examine the hallmark characteristics of ovarian CSC and shared by all stem cells, as potential for self-renewal, lineage differentiation and homeostatic control.

The outlook for patients with ovarian cancer may be markedly improved by identifying disease-specific CSCs which are relevant to the development of each subtype of cancer. The involvement of CSCs in chemoresistance and recurrence opens a new avenue to develop new CSC-specific drug-delivery conjugates in the form of aptamers, differentiating agents, miRNA mimics or targeting peptides/nucleotides. In addition, the application of personalized medicine in the form of a genomic signature (DNA or RNA), even though not yet standardized and integrated into the health system for clinical consideration, may facilitate individual-specific drug and dose selection resulting in better ovarian cancer diagnosis and prognosis.

The rapid increase in our understanding of molecular processes that regulate cancer signatures has raised an equally strong desire to eradicate EOC before the resistance, or relapse that continue to worsen survival data of this disease. Multiple ovarian histophenotypes and the possible sites of disease origin, together with the potential for differential hierarchal contributions of multiple CSCs populations, represent significant challenges for the identification, functional characterization and therapeutic targeting of ovarian CSC.

## Competing interests

The authors declare that they have no competing interests.

## Authors’ contributions

FT and AP were the main authors of the manuscript; LR and MS collected and studied the bibliography; PV participated in the sequence alignment and drafted the manuscript; GL corrected the language form; ST drafted the article and revised it critically for important intellectual content. All authors read and approved the final manuscript.
